# Medical Practitioners and the Midwives Act

**Published:** 1907-03-02

**Authors:** 


### Medical Practitioners and the Midwives Act.

The historical account of the evolution of the official midwife, which has recently been put on record by Dr. C. J. Cullingworth, will revive the memories of some acute and comparatively recent controversies. Opinion in the medical profession was sharply divided in reference to the policy which led up to the Midwives Act of 1902, and also in regard to the corporate action of the Obstetrical Society in examining and certifying midwives. It will be remembered that at one time the Society's action came under the notice of the General Medical Council, and that the Council pronounced condemnation in no uncertain terms. The Central Midwives Board is now the sole licensing authority by which the examination and certification of midwives is conducted. Similarly, the Midwives Act is now an accomplished fact, and even those who object to it must realise that it has come to stay; the legally recognised widwife must be accepted as one of the permanent factors of the situation.

Looking to the future it is probably at present too early to advance a complete criticism of the operation of the Act which became part of the law of the land in 1902. Many experiences under its provisions have provoked complaint and discontent, and there can be little doubt that sooner or later the whole question will come under Parliamentary review. In the meantime it is essential to emphasise the injustice which the present position entails on medical practitioners called upon to attend women in accordance with the provisions of the existing Act. As has been pointed out again and again, no arrangement is made for the payment of medical services rendered under these conditions. The midwife, in the presence of any one of a number of contingencies, must, according to the terms of the law, advise that a medical practitioner be summoned, and must decline further services unless this advice is accepted. Whatever may be the exact technical position of the summons which in these circumstances reaches the practitioner, it is in effect and substance a demand advanced under the provisions of an Act of Parliament, and most certainly it ought to carry a guarantee of payment on the part of some public organisation, say the municipal or local health authorities. Whether these authorities should or should not endeavour to recover from the patient the fee thus paid is a social and economic question with which the medical profession in its corporate capacity has no immediate concern. The position of the practitioner is that he is called upon to render services by an authority which finds its sanction in the voice of the community as expressed in an Act of Parliament, and that his fee ought to be provided by those who have called this authority into existence. As it is, the practitioner is placed in the difficult position either of having to refuse to attend, or to attend with little or no prospect of being paid for his services. The summons, when it arrives, is in itself a proof that a critical position has arisen, and the natural and humane instincts of every medical practitioner prompt him to render aid without a too careful consideration of the question of fee. Yet such a course may mean not only the absence of remuneration, but also the neglect of his own patients and practice and the risk, in the case of puerperal fever, of inability to accept private midwifery engagements for a considerable period of time. The community has no right to place these demands on any of its members, and still less to advance them without a guarantee that they shall be adequately remunerated. Of course, it is true that a medical man is not legally compelled to attend to the summons of a midwife. We not only admit this, but we say that there are circumstances in which, in justice both to himself and his patients, he ought to refuse such a summons. Yet though choice rests with the practitioner, there are conditions under which refusal is almost impossible, except at the risk of public odium. And in either event the essential position is not modified.

There is another aspect of the situation which the medical profession must keep in view. It is certain that many members of the profession do not desire, altogether apart from the question of fee, to be called to cases under the Midwives Act. Others may be quite willing to accept these cases. There may thus be the necessity of arranging in each locality a list of practitioners willing to work under the Act, and the midwives might be compelled to summons these according to a system of rotation. Possibly some may consider that the best thing is to appoint in each district one medical officer as a kind of municipal accoucheur and to restrict the practice of such an officer to work under the Midwives Act. Here there is room for some difference of opinion, but there is no such possibility in reference to the main position discussed in this article.

